# The Antibodiome—Mapping the Humoral Immune Response to HIV

**DOI:** 10.1007/s11904-019-00432-x

**Published:** 2019-03-22

**Authors:** Audrey L. Butler, Stephanie Fischinger, Galit Alter

**Affiliations:** The Ragon Institute of MGH, MIT, and Harvard, 400 Technology Square, Cambridge, MA 02139 USA

**Keywords:** Antibodies, HIV-1, Vaccination, Natural HIV control, Innate immunity

## Abstract

**Purpose of Review:**

The design of an HIV vaccine remains an elusive but top priority. Data from the non-human primate model and the first moderately protective HIV vaccine trial (RV144) point to a role for qualitative changes in humoral immune functions in protection from infection. Here, we review the current understanding of the antibody response throughout HIV infection, the known correlates of protection, and current strategies to manipulate antibodies to put an end to the epidemic.

**Recent Findings:**

Recent studies point to innate immune-recruiting antibody function in preventing infection as well as controlling viremia following infection. These data have begun to inform next-generation design of HIV vaccines and antibody therapies by uncovering new viral targets and antibody architectures to improve potency and breadth.

**Summary:**

Emerging data illustrate a role for innate immune recruiting-antibodies in conferring protection against HIV infection as well as promoting viral control and clearance, offering an unprecedented opportunity to modulate and improve antibody function to fight HIV more effectively.

## Introduction

Empirical vaccine design has led to the generation of clinically approved vaccines against 26 pathogens, yet similar approaches have failed against HIV [1]. This is due in part to the uniquely high mutation rate of HIV and the low density of envelope (Env) protein on the viral surface, which together restrict the evolution of neutralizing antibodies. Only a limited number of sites of neutralizing vulnerability have been defined on the HIV Env protein, which require highly specialized attachment footprints and angles of attack for antibody-mediated neutralization [2, 3]. This knowledge has driven vaccine design efforts to focus on the development of immunogens that display only minimal scaffolded surfaces that solely present the site of neutralization attack [4]. Additionally, methods have been developed to deliver sequential Env immunogens through vaccination. This approach is meant to direct the evolution of humoral immunity to HIV epitopes that render the virus more vulnerable to neutralization [5]. These strategies, among others, have emerged from the hypothesis that neutralizing antibodies are essential for global protection against HIV. However, accumulating data from human and non-human primate (NHP) vaccine studies have systematically challenged this dogma by pointing to non-neutralizing functional antibodies as correlates of protection [6, 7].

The first large-scale HIV vaccine trials starting in the late 1980s aimed to induce neutralizing antibodies against the HIV envelope (Env) protein [8]. Using a recombinant gp160 protein antigen for immunization, the first vaccine instead elicited high titer binding antibodies in the absence of significant neutralization, and no protection was observed in the trial [9]. The next two large trials VAX003 and VAX004 induced neutralizing antibodies but afforded no significant protection [10]. When vaccination could not drive the development of robust neutralizing antibody responses to confer protection, the field shifted focus to emerging data indicating a critical role for T cells in viral control [11]. This inspired the testing of a T cell–focused vaccine (adenovirus 5—Ad5) strategy. Unfortunately, the Ad5-based vaccine study was halted prematurely due to evidence of increased risk of HIV acquisition among vaccinees [12], which was linked to enhanced T cell activation particularly in the gastrointestinal tract [13]. These data hinted that T cell vaccines may be insufficient to drive protection from infection; although it was unclear whether the lack of efficacy was due to the specific vector used or if results would generalize across all vectored T cell–inducing approaches [14].

Concomitantly, a viral vector prime, protein boost strategy was underway using a pox virus prime (ALVAC) and a recombinant Env boost, which resulted in a modest vaccine efficacy of 31.2% [6, 15, 16]. Importantly, this RV144 trial provided the first evidence of vaccine-mediated protection against HIV in the absence of responses originally hypothesized to be correlates of immunity: neutralizing antibodies and cytotoxic T cell responses. Instead, this protection was linked to the induction of non-neutralizing IgG1 antibodies targeting the variable loop 2 capable of driving antibody-dependent cellular cytotoxicity [15]. However, this same strategy using a different viral vector for prime/boost, DNA/Ad5 (also aimed at inducing both T and B cell responses), resulted in no evidence of protective immunity [12], suggesting that the quality of the prime/boost may be essential for tuning vaccine-induced immunity for protection. Collectively, these data clearly indicated that (1) protection against HIV may be achievable through vaccination, (2) protection does not require neutralizing antibodies or cytotoxic T cells, and (3) qualitatively superior functional antibodies may be essential for protection.

In addition to their role in protection, amassing evidence suggests that antibodies may also contribute to natural control of HIV. Specifically, while only a fraction of spontaneous HIV controllers harbor broad T cell immunity [17], a large proportion of controllers possess highly functional antibodies capable of inducing potent innate antiviral responses [18]. Studies have linked higher levels of such antibody effector functions to lower viral loads [19] and slower disease progression [20]. Moreover, functional non-neutralizing antibodies have been shown to drive antiviral control when induced prior to challenge [21]. Thus, defining the specific antibody effector functions that track with enhanced viral control may provide valuable insights that can be applied to vaccine design not only for prevention of infection but also for therapeutic control of the viral reservoir.

In this review, we summarize the knowledge related to the evolution of functional humoral immunity in HIV infection and the correlates of both spontaneous control and vaccine-conferred protection from infection. Finally, we explore how dissecting protective profiles can inform the design of improved HIV vaccines and monoclonal antibody therapies.

## Targets of Protective Antibodies

For most diseases, vaccines are designed to induce antibody responses against the most abundant and immunogenic surface antigen(s) [22]. This is often informed by the natural antibody-specificities associated with pathogen control or containment. For HIV, only one antigen—Env—composed of gp120 and gp41 subunits, is expressed on the viral surface at low densities (only 7–14 Env trimers/virus) [23]. Moreover, the Env antigen can adopt several states including (1) a trimer (3 gp120 and 3 gp41 units) required for infection, (2) non-infectious monomers (gp120/gp41), and (3) unassembled gp41 “stumps” [24]. Since HIV steals its membrane envelope from the host cell, most antigens on the surface of the infecting virion are human-derived. This renders the virion nearly invisible to the immune system. While it is clear that the “trimer” represents the key target for neutralizing antibodies [25, 26], it is unclear what form of the antigen is presented by infected cells to stimulate the killing mediated by protective functional antibodies.

In order to address this question, discussions have emerged concerning which antigenic targets are most relevant for targeting by non-neutralizing killer antibodies. Upon CD4+ T cell infection, expression of the HIV *Nef* gene product drives the rapid downregulation of CD4 from the surface of the infected cell [27]. This permits newly produced HIV virions, decorated in Env proteins, to avoid binding –in-cis—to CD4 (the HIV Env receptor), allowing the virus to successfully leave the cell and bud off [27]. The elimination of in-cis binding also protects the infected cell from ADCC activity [28]. As a result, several groups have argued that killing cells which are actively producing virus is likely to be focused on cells that no longer express CD4. More critically, several ADCC-inducing antibody epitopes have been identified that bind to unique sites that are unmasked upon HIV Env binding to CD4. These CD4-inducible (CD4i) epitopes are not exposed on the native trimer present on the virus until after Env binding to CD4 [29]. Given the low level of CD4-bound Env on productively infected cells, producing virus, it is argued that these ADCC antibodies may instead target uninfected bystander cells that have incidentally picked up Env on CD4. Since several in vitro antibody-effector assays either capture HIV Env on the surface of CD4-expressing cells or include cells at different stages of CD4-downregulation, concerns have emerged related to the interpretation of “relevant” functional correlates of protection.

This important question remains largely unanswered but may be less concerning in the setting of a protective vaccine response. In this case, few cells are successfully infected [30], and cells that are infected are thought to exist in limited foci of infection. These foci likely contain cells at multiple stages of infection with variable amounts of surface-expressed CD4 and variable levels of virus production. Antibodies specific for CD4-bound HIV Env that can destroy cells in different stages of infection, despite changes in epitope availability and amount of CD4-bound targets, are likely to confer the greatest level of protection against HIV. However, death of bystander cells is one potential consequence of employing antibodies targeting cells that continue to express CD4. While this phenomenon is clearly undesirable in the setting of high-level disseminated HIV infection, the elimination of a few non-infected CD4-expressing bystander cells may be acceptable if more non-specific antibody-mediated killing contributes to the effective deletion of all originally infected cells within limited foci of infection. This may also be true in the context of HIV eradication, where infected cells may not be actively producing virus but may still express some CD4 on the surface. Moreover, CD4i-specific antibodies are abundant in HIV controllers, who do not exhibit a significant decline in CD4+ T cell numbers [31]. This suggests CD4-targeted antibody responses do not cause immunopathology and instead are enriched in a setting where effective viral control is maintained. Therefore, it is possible that polyclonal pools of antibodies that target cells downregulating CD4 to varying degrees may be most desirable to ensure the effective elimination of cells at both early and late stages of HIV infection during the acute phase. While this represents the ideal case, efforts to define the specificities of protective functional antibodies are still underway.

Despite these unknowns, emerging data continue to uncover details about the early development of functional antibodies that predict antiviral control and disease progression. Additionally, given the field’s new appreciation for the evolution of neutralizing antibodies in a significant proportion of infected individuals, studies investigating the natural progression of protective humoral immune responses over the course of HIV infection in different populations continue to provide critical clues for the development of vaccines able to leverage protective functions of antibodies.

### Antibody Functional Evolution From Acute HIV Infection

**Fig. 1:**
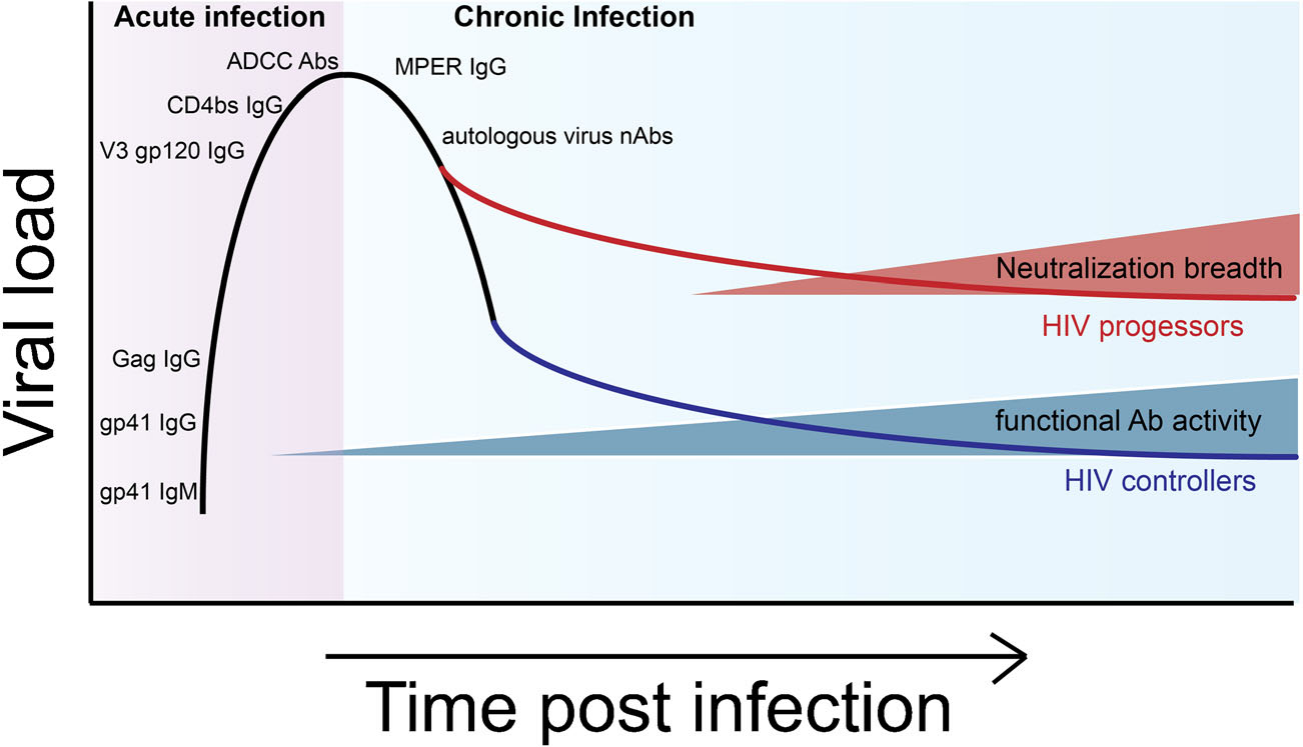
Humoral immunity timeline in HIV. During the first weeks of acute infection, HIV envelope-specific IgM and IgG antibodies are produced sequentially to a number of epitopes (gp41, gp120, V3 loop, CD binding site, and MPER) and are non-neutralizing but capable of inducing Fc-mediated functions, such as antibody-dependent cellular cytotoxicity (ADCC) by natural killer (NK) cells. The first neutralizing antibody responses appear after months of infection and are specific to autologous viral strains. Over the following years, some individuals spontaneously control infection. These individuals harbor innate immune-recruiting antibodies. Broadly neutralizing antibody responses, conversely, evolve largely in individuals who fail to control infection

The acute window of HIV infection occurs in the first 2–4 weeks following acquisition. During this time, diagnostic tests often fail to detect infection, as antibodies are still developing during this phase. Importantly, the HIV Env gp160 protein is cleaved intracellularly into two gene products: gp41 that forms the transmembrane region, co-receptor binder, and fusion machinery; and gp120, the extracellular envelope region involved in initial CD4 binding. Although gp41 is more recessed on the viral surface, antibody responses to this epitope evolve first (Fig. 1). Gp41 responses are often detected in the first 1–2 weeks of infection [32] and are thought to arise earliest through the recruitment of pre-existing microbiome memory B cells specific for antigen epitopes that overlap with those found on gp41 [33]. Gag-specific IgG antibodies (p24, p55), used diagnostically, appear 2–3 weeks after infection, followed by gp120-specific antibodies [26]. Interestingly, epitope-specific recognition across HIV Env also emerges over time. V3-loop-specific [26, 34] and CD4 binding site antibodies appear after 1 month of infection [35], and although rare, recessed membrane-proximal external region (MPER)-specific gp41 IgG antibodies arise after 5–10 weeks of infection [36] (Fig. 1).

While early antibodies do not neutralize HIV [34], they still contribute to antiviral immunity by recruiting innate immune effector functions, such as natural killer (NK) cell induced antibody-dependent cellular cytotoxicity (ADCC) [37]. Specifically, evidence of in vitro ADCC activity during acute HIV infection was demonstrated when patient plasma and purified IgG were shown to hamper viral replication in the presence of NK cells from healthy donors [38]. Substantial inhibition of viral replication was observed when plasma from acutely infected patients was combined with infected target cells in the presence of NK cells but not in the absence of these effector cells, suggesting antibodies play an important role in controlling infection in the absence of neutralizing activity. Importantly, this innate immune recruiting activity was found to be broadly reactive and inversely correlated with plasma viremia during acute infection. This indicated a potential role for these antibodies in establishing set point viremia. Bolstering these in vitro data, studies have identified antibodies capable of mediating ADCC in patients with lower viral set points across the globe [39–42].

Studies highlighting that robust early ADCC activity predicts slower disease progression throughout chronic infection [37] have further supported the protective role for these antibody functions in viral clearance. However, whether these antibody functions that arise in parallel to T cell responses work in concert with T cell immunity, proposed to be key a mediator of early viral control [43], remains unclear. Yet, vaccination resulting in the induction ADCC prior to infection has been shown to reduce viremia in non-human primates [44], providing direct evidence for the role of ADCC-inducing antibodies in antiviral immunity. Analyses of the evolution of these functional antibodies in acutely infected patients have pointed to the development of early ADCC responses against the conformationally intact Env trimer on the virion surface which is followed by the expansion of ADCC-inducing antibodies against linear epitopes [42]. Interestingly, these NK cell-recruiting antibodies were shown to evolve reciprocally with that of neutralizing antibodies, suggesting mutually exclusive paths of antibody effector development [45]. These data potentially argue for an important shift in ADCC-inducing antibody specificity, with qualitatively superior virus-sensing antibodies early in disease.

### Chronic HIV Infection

Following the first month of infection, neutralizing antibodies (nAbs) specific for the autologous infecting HIV strain appear, marking the transition to chronic infection [26]. The virus rapidly mutates in response to the immune pressure exerted by these nAbs, and in turn, the host develops new autologous nAbs to the evolving virus. This cycle continuously occurs, with the virus consistently remaining one step ahead of the neutralizing antibody response [46]. Even after a few months, newly generated nAbs continue to exhibit limited potency and show restricted specificities in about 80% of patients. Over years of chronic infection, in the setting of a perpetually mutating virus, these neutralizing antibody responses diversify, and in a fraction of infected individuals, gain the capacity to neutralize heterologous strains of the virus [47]. In a large African sero-surveillance study, antibodies able to neutralize multiple viral strains were observed in 34% of volunteers [48]. Additional studies have highlighted the presence of cross-reactive neutralizing antibodies in approximately 25% of infected individuals [32, 49, 50]. However, broadly neutralizing antibodies (bNAbs) that can neutralize global variants across HIV clades develop in only about 1% of infected individuals, otherwise known as “elite” neutralizers [48]. The detection of neutralization breadth across populations around the world shows that the evolution of bNAbs, while rare, is immunologically tolerated and possible.

While breadth of neutralization evolves in a significant proportion of the infected individuals, several years of infection are required to acquire these protective immune responses. Specifically, evolution of breadth of neutralization occurs over the first 1–3 years of infection, with the percentage of individuals’ neutralizing responses against multiple strains and clades increasing from less than 30 to 75% [51]. Epidemiologic analyses aimed at identifying the clinical characteristics associated with developing neutralizing antibody breadth have identified that higher viral load set point, increased viral diversity, elevated CD8:CD4 lymphocyte numbers, increased HIV-specific B cell frequencies, greater immune activation, higher Env-specific IgG titers, and early envelope diversity are all associated with the evolution of antibody breadth [32, 51–53].

In addition to these clinical and immunological correlates, studies have clearly demonstrated an intimate interaction between viral mutational events and neutralizing antibody progression [49, 50]. Perpetual waves of HIV mutation forced by nAbs throughout infection drive the production of new clonal antibody repertoires with enhanced affinity and avidity as HIV-specific B cells cycle through many rounds of selection. However, HIV subverts immunity so successfully that this immune pressure has limited antiviral impact [46]. This is further evidenced by the enrichment of broadly neutralizing antibody responses among individuals with high viral loads, high levels of immune activation, and low CD4+ T cell counts [54, 55]. Yet, while these nAb responses fail to control viremia in humans over years of infection, the passive transfer of human bNAbs into non-human primates (NHPs) confers robust protection from viral challenge [56–58]. This highlights the possibility that a vaccine able to generate these responses prior to viral exposure could effectively prevent infection in humans, as the appropriate antibodies would be present before HIV escape could occur.

Similarly to neutralizing antibodies, non-neutralizing antibodies have been observed to influence viral escape and mutation [59, 60]. However, unlike bNAbs, non-neutralizing antibodies are largely enriched among individuals with lower viral set points [18, 37, 61–65, 66•, 67, 68], suggesting that these responses may have a more significant impact on diversified viral populations. These data may also imply that HIV escape mechanisms are insufficient to evade these functional antibody responses. Supporting the unique role for functional antibodies in chronic infection, longitudinal analysis in rhesus macaques showed sustained plasma ADCC activity, resulting in higher CD4 T cell counts and delayed progression to AIDS as compared to other NHPs that experienced waning titers of ADCC-mediating activity early after viral inoculation [69]. These data strongly argue that ADCC and potentially other effector functions may play a more critical role than neutralization in antiviral control throughout chronic infection.

## Correlates of Spontaneous HIV Control

One of the characteristics of chronic HIV infection is the heterogeneity in disease progression rates across HIV-infected populations [70, 71]. Specifically, viral load set point is a strong predictor of the rate of progression to AIDS. Subjects with high viral loads progress more rapidly than those who spontaneously control viral replication to low levels, known as controllers or long-term non-progressors (LTNP) [70, 71]. Intriguingly, a small subset of HIV-infected individuals, referred to as elite controllers (ECs), maintain stable CD4+ T cell counts and virtually undetectable levels of viremia [72]. Elite controllers exhibit reduced HIV-specific T cell activation and maintain polyfunctional T cell responses [73].

Efforts to define the mechanism(s) that may account for viral control identified an enrichment of genome wide-associated single nucleotide polymorphisms (SNPs) within the major histocompatibility complex (MHC) [74] in controllers. These SNPs, localized to specific class I MHC-B and MHC-C alleles, were associated with the presentation of more conserved HIV-derived peptides as well as improved interactions with NK cells, resulting in more effective killing of HIV-infected cells [75]. However, these alleles are present in only a fraction of ECs [17] and these SNPs account for just 15% of the variation in viral set point [74]. This suggests other features of the host/pathogen interaction are critical for antiviral control at a global level. Additional explanations for suppression have argued that some controllers are infected with attenuated viral strains, enabling these individuals to control the virus more effectively [76]. However, most controllers are infected with replication-competent virus [77].

**Fig. 2:**
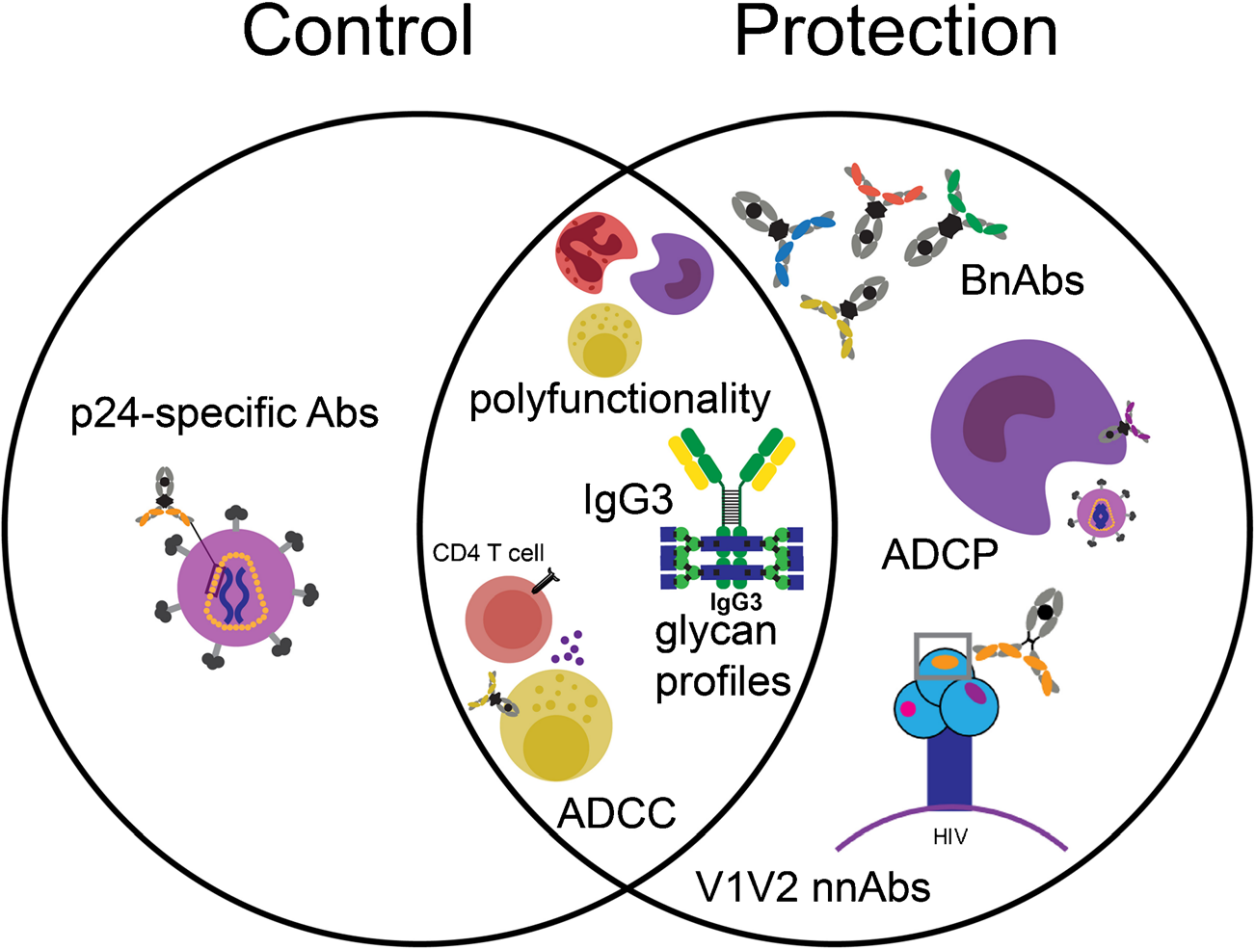
Known correlates of protection and viral control. Polyfunctional HIV-specific antibody responses (able to recruit multiple innate immune effector cell populations), higher HIV-specific IgG3 antibodies, unique HIV-specific antibody glycan profiles, and elevated ADCC activity are enriched in both spontaneous controllers and in animals or humans protected from infection. Controllers also exhibit elevated levels of p24-specific antibodies. Additionally, protected vaccinated humans and NHPs harbor elevated V1V2-specific antibodies and antibodies able to drive antibody-dependent cellular phagocytosis (ADCP). Finally, while not enriched in naturally protected individuals, the administration of broadly neutralizing antibodies (bNAbs) can confer protection against infection. Thus, many shared, but some unique humoral profiles, are associated with protection from infection and control of viremia

To understand the factors that contribute to viral control beyond genetics and viral infectivity, the field has sought to identify additional immune responses that may be uniquely enriched in ECs (Fig. 2). Analyses of the HIV-specific humoral immune responses in this unique patient population have shown an enrichment of ADCC-inducing antibodies [18, 37, 61–65, 66•, 67, 68]. Specifically, ECs and LTNPs appear to have enhanced ADCC response and target both the viral Env and regulatory/accessory viral proteins, such as Vpu, that are not observed in progressors [66•]. Moreover, ADCC activity against the structural Env V3 loop region as well as against Gag and Tat proteins is disproportionately higher in controllers [78, 79]. Importantly, although abundant, ADCC is not the only innate effector function induced at high levels in controllers. Studies have clearly illustrated that unlike the general HIV-positive population, elite controllers produce a highly polyfunctional humoral response [64]. This drives the generation of HIV Env-specific antibodies able to access a broader array of innate immune functions including ADCC via NK cells, antibody-dependent cellular phagocytosis (ADCP), antibody-dependent complement deposition (ADCD), and antibody-dependent neutrophil phagocytosis (ADNP). This enhanced polyfunctionality is associated with an enrichment of more functional HIV-specific antibody subclass profiles in controllers. This is marked by elevated levels of IgG3 antibodies, known to have enhanced affinity for Fc-receptors and complement to enable the more effective induction of innate immune effector functions [80, 81].

Despite robust evidence supporting a role for ADCC in natural antiviral control, functional antibodies have not yet been shown to provide protection from infection when transferred into NHPs before viral challenge. For example, transfer of polyclonal antibodies from ECs had no impact on HIV acquisition in NHPs. However, titers of these transferred antibodies were associated with a trend towards lower levels of viremia in animal plasma [82]. It remains unclear if this lack of protection was due to infusion of low titers of antibodies incapable of forming the types of immune complexes needed to drive innate immune effector function. Although effector functions may not be primary drivers of protection, they are still known to have therapeutic value. Passive transfer of monoclonal bNAbs into chronically infected NHPs and humans has had profound therapeutic effects on suppressing viremia [58, 83••]. In NHPs, this has been linked to the magnitude of antibody-mediated activation of NK cells and neutrophils [84], suggesting that Fc-functionality is key to antiviral control and clearance. While passive transfer studies have yet to causally link ADCC or other innate immune effector functions with protection from infection, these non-neutralizing antibody-mediating responses still appear to be critical predictors of both viral set point and viral control following immunization.

Observations of unique functional responses in controllers have prompted deeper investigation into the specific mechanisms that controllers selectively evolve to access broader innate immune activity. Beyond the role of different antibody subclasses in driving enhanced antibody effector function, antibody Fc-glycosylation is also a key modulator of Fc-receptor and complement activation [85, 86]. Interestingly, early studies in HIV-infected patient populations noted elevated levels of agalactosylated antibodies, associated with autoimmune disease [87], in HIV-infected patient populations [88]. Follow-up analyses pointed to an enrichment of inflammatory agalactosylated antibodies among controllers [89] despite lower immune activation [73, 89]. Moreover, controllers appear to skew glycosylation of HIV-specific antibodies to enhance antibody effector function [89]. Specifically, controllers selectively generate Env-specific antibodies with less galactose and higher levels of *n*-acetyl glucosamine residues, thought to be key for promoting enhanced binding to FcγR3a found on NK cells. These data provide evidence of two mechanisms by which controllers selectively enhance Fc-effector activity, via both (1) the selection of more functional antibody subclasses and (2) the generation of antibodies with Fc-glycans engendering enhanced affinity for specific Fc-receptors.

While ADCC activity trends higher in controllers [18, 37, 61–68], these individuals do not necessarily elicit higher titers of total or subclass-specific antibody responses [80]. Yet interestingly, they consistently possess higher levels of p24-specific IgG [80, 90, 91] (Fig. 2). However, it is unclear if these higher p24-specific responses contribute directly to antiviral control, due to the negligible level of p24 expressed on the surface of infected cells or simply reflect the conservation of a more functional immune response. Nevertheless, this increase in p24 antibodies is characteristic of controllers worldwide [92].

Conversely, neutralizing antibodies are largely found in equal or lower frequencies in controllers than in the overall HIV-positive population [93]. Large cross-cohort analyses have linked the evolution of neutralizing antibodies with higher viral loads and lower CD4+ T cell counts [55, 93], which supports their development in HIV progressors rather than in controllers. Yet, some of the field’s most promising broadly neutralizing antibodies have been cloned from controllers [94]. How controllers can develop neutralizing antibodies in the absence of high levels of antigen exposure and viral evolution is perplexing. Recent data point to the evolution of bNAbs in controllers who exhibit a unique inflammatory signature marked by elevated levels of CXCL13, TNF, RANTES, IP10, and sCD40L [95]. Interestingly, this unique inflammatory profile was linked to detectable viral RNA, suggesting that the controllers who evolve neutralizing antibody breadth experience constant antigenic exposure, potentially required to drive the development of broadly neutralizing antibodies. These data suggest that vaccine strategies aimed at eliciting bNAbs will require persistent antigenic exposure to drive ample B cell evolution and selection.

Overall, clues from natural infection point to a critical enrichment of functional antibodies in patients who spontaneously control HIV. These data also point to unique approaches to co-evolve nAb and non-nAb activities to leverage both ends of the antibody in HIV prevention.

## Correlates of Protection From Infection

Correlates of protection from infection have emerged from active and passive immunization studies in humans and non-human primates (NHPs) [96]. Remarkably, the passive transfer of neutralizing antibodies (nAbs) into NHPs has consistently afforded protection even at low doses [57, 97–102]. In contrast, passive transfer of functional non-neutralizing antibodies has not demonstrated the same level of protection. For example, in a study where pooled IgG from Simian immunodeficiency virus (SIV)-infected macaques was infused into uninfected monkeys preceding HIV challenge, non-neutralizing antibodies failed to provide sterilizing protection. However, infected monkeys still showed slower progression to AIDS as well as a decrease in plasma viremia, which was linked to antibody-dependent effector functions [103].

Active immunization in both humans and NHPs repeatedly fail to induce broadly neutralizing antibodies [104]. Still, protection from infection has been observed across multiple NHP studies and in one human vaccine trial [15, 96, 105–112]. In these cases, protection was linked to the induction of specific Fc-effector profiles. Human correlates of immunity have largely emerged from the RV144 vaccine trial, which showed a moderate level of protection in approximately 31% of vaccines [15]. Correlates of protective immunity included V1/V2-specific IgG1 and IgG3 responses [113], low IgA responses [6], and higher levels of ADCC activity [15] (Fig. 2). In addition to ADCC, V1/V2-specific complement activating serum IgG was shown to correlate with reduced HIV infection in the RV144 vaccine trial [114]. Moreover, vaccination in RV144 led to the induction of polyfunctional antibody effector profiles [16] similar to those observed in spontaneous controllers of HIV [64], which was not the case in trials that did not elicit protective responses. This revealed common Fc-effector correlates across natural and vaccine-induced protection.

While only one HIV vaccine has shown promise for protection in humans, a variety of immunization regimens have prevented viral acquisition in NHPs [96, 105–112]. Protection from infection ranging from 20 to 66% has been found to be associated with antibody binding to HIV-infected cells, ADCC, antibody-induced activation of MIP-1β in NK cells, Env-binding antibodies, V2-specific antibodies, polyfunctional antibodies, and ADCP. For example, using adenovirus 26 (Ad26) and protein boosting, 50% and 66% protection was observed against SIV and SHIV challenge, respectively, in the absence of neutralizing antibody activity [105, 115]. Analysis of correlates of immunity identified antibody titer and antibody Fc-effector function as primary mediators of protection. Similarly, multiple antibody functions were linked to protection following both poxviral vector (ALVAC) prime/protein boosting [111] and administration of DNA/Ad5 [110, 116••]. Specifically, in the latter DNA/Ad5 study, phagocytosis emerged as a key correlate. This was mediated by distinct innate immune effector cells and depended on the route of immunization [116••]. Specifically, intramuscular protection was tightly linked to monocyte-mediated phagocytosis, while mucosal vaccination was tied to neutrophil phagocytosis with a specific role for IgA. In both vaccine arms, protection and function involved antibody binding to FcγR2. The same Fc-phagocytic/FcγR2 correlates predicted protection in the ALVAC/protein vaccine study [110], and ADCP has been associated with protection from infection following Ad26/protein immunization as well [105]. These results illustrate the universality of these protective correlates across different vaccine platforms in NHPs. Therefore, accumulating data indicate a critical role for phagocytosis, mediated by a variety of cells, through FcγR2 signaling, in protection against infection.

Along these lines, passive transfer of broadly neutralizing antibodies (bNAbs) also supports the importance of Fc-effector function in protection. Using a mouse model of HIV infection, some, but not all, bNAbs required Fc-effector function to confer protection from infection [117]. Moreover, the passive transfer of the bNAb PGT121 in NHPs resulted in robust non-sterilizing protection from infection, linked to innate immune activation [118••] even at distal sites days after viral challenge. Notably, eliminating Fc-effector function from bNAbs compromised protection from NHP infection [119]. However, specific Fc-mutations meant to enhance ADCC activity did not improve the protective activity of broadly neutralizing antibodies [120]. These data highlight that even broadly neutralizing antibodies may require Fc-mediated effector activity, but functions beyond ADCC may be required for protection.

Thus, like correlates of spontaneous control, insights gleaned from active and passive immunization strategies collectively demonstrate the need to harness both ends of the antibody to fully capitalize on the protective nature of the humoral immune response and prevent HIV infections at a global level.

## Opportunities for the Future

The vast knowledge acquired by the HIV field in the last three decades provides exciting opportunities to guide the design of protective vaccines and therapeutics. These include approaches to leverage both ends of the antibody to enhance both the blockade and killing of the virus and virally infected cells. Despite the successes of pre-exposure prophylaxis (PrEP) and anti-retroviral therapy (ART), a globally protective vaccine remains the simplest and most effective approach to end the HIV epidemic. Adherence to a daily regimen is necessary for the effectiveness of both PrEP and ART, whereas the development and deployment of a durable vaccine is likely to reach a larger fraction of the globe. However, there are still many obstacles to overcome due to the complexity of HIV infection, global diversity, and the subsequent highly heterogenous immune response. Nevertheless, defining correlates of spontaneous HIV control and protection from infection has brought us many steps closer to achieving better control of the disease across the globe and reaching the goal of vaccine design that may leverage these natural immune responses to gain control over the virus. While empirical vaccine design approaches have failed in HIV for the past four decades, emerging correlate-inspired vaccines and therapeutics are certain to revolutionize our fight against HIV.
